# Retraction: Case report: A rare case of tumor-to-tumor metastasis: metastatic lobular breast carcinoma to clear cell renal cell carcinoma

**DOI:** 10.3389/pore.2024.1611784

**Published:** 2024-04-05

**Authors:** Pathology and Oncology Research Editorial Office

Following publication, the authors contacted the Editorial Office stating that concerns were raised regarding the scientific validity of the article. In particular, the authors stated that the case presented in the article was not adequate to strongly support their conclusion. Additionally, they stated that the discussion section required further, updated literature, and that the authors were planning to perform additional experiments to support their conclusions.

The authors failed to provide any further explanation since their initial correspondence. As a result, and following an investigation conducted in accordance with Pathology and Oncology Research policies by the editorial board, the conclusions of the article have been deemed unreliable and the article has been retracted.

This retraction was approved by the Editor-in-Chief of Pathology and Oncology Research. The authors received communication regarding the retraction but did not finalize their agreement. The communication has been recorded by the publisher.

